# Health Insurance Mandates for Nonpharmacological Pain Treatments in 7 US States

**DOI:** 10.1001/jamanetworkopen.2024.5737

**Published:** 2024-04-10

**Authors:** Taylor Nadine Onstott, Samantha Hurst, Richard Kronick, An-Chi Tsou, Erik Groessl, Sara B. McMenamin

**Affiliations:** 1 Herbert Wertheim School of Public Health and Human Longevity Science, UC San Diego, La Jolla, California; 2 California Health Benefits Review Program, Berkeley

## Abstract

This cross-sectional study examines the extent to which states have introduced or enacted mandates for coverage of nonpharmacological pain treatments and characterizes the variation in such mandates.

## Introduction

There has been recent interest by legislative bodies in increasing access to nonpharmacological pain therapies as a nonopioid alternative for pain. Prominent clinical practice guidelines recommend the use of nonpharmacological pain treatments such as acupuncture, cognitive behavioral therapy, psychotherapy, chiropractic care, massage, osteopathic manipulation, and physical therapy.^1,2,3,4^ Despite these recommendations, health insurance coverage for nonpharmacological pain treatments among both commercial and public plans, such as Medicare and Medicaid, is inconsistent.^5^ This study (1) documents the extent to which states have introduced or enacted mandates for coverage of nonpharmacological pain treatments and (2) describes and characterizes variation in such mandates. The findings from this study may help inform policy makers and relevant stakeholders considering legislation related to nonpharmacological pain treatments at the state and federal level.

## Methods

This cross-sectional study used systematic document collection and performed qualitative analysis. Two online legislative tracking websites (PoliticoPro and LegiScan) were searched in November 2022 and again in August 2023 to identify legislation that either (1) encouraged or mandated coverage of nonpharmacological pain treatments or (2) specified the terms of coverage (eg, cost sharing, prior authorization, reimbursable clinicians) for nonpharmacological pain treatments (eAppendix in Supplement 1). We characterized relevant policies according to 2 characteristics identified a priori: (1) coverage and (2) coverage limitations and 3 new codes that arose from the analysis: (1) types of pain, (2) evidence-based coverage, and (3) essential health benefits (EHB)–related provisions. These study methods followed the STROBE reporting guideline. Institutional review board approval and informed consent were not required because the research did not involve human participants as defined by 45 CFR §46.102. Statistical analysis was performed using Excel version 16.83 (Microsoft) from January 2023 to March 2023.

## Results

This study’s search yielded 13 policies introduced in 7 different US states between January 2019 and August 2023, of which 2 were enacted, 3 were in progress, 1 was vetoed, and 7 were not enacted (Table 1). Five states proposed legislation mandating coverage of nonpharmacological pain treatments, yet none enacted such legislation. California enacted legislation to encourage (but not require) coverage. Only 1 state (Kentucky) included Medicaid enrollees in their proposed coverage mandate legislation. Five states also proposed legislation related to permitted limitations to nonpharmacological pain treatment coverage, with 4 proposing and 1 (Colorado) enacting legislation requiring cost-sharing for such coverage to be at parity with primary care visits. Other elements of proposed coverage varied. Acupuncture and chiropractic care were the treatments most consistently mandated; in addition, 4 states required evidence-based coverage, 3 states included EHB-related provisions to protect states from incurring additional cost, and 2 states included language that limited coverage to chronic pain (Table 2).

**Table 1.** Nonpharmacological Pain Treatment Benefit Mandates by State, Year Introduced, Bill Number and Status, Content, and Eligible Population

State	Year	Bill No. (status as of 8/2023)	Content^a^	Eligible population
California^b^	2022	AB 2585 (enacted, effective Jan 1, 2023)	Encourages coverage	References the whole health care system and all clinicians.
Colorado^c^	2020	HB 20-1085 (vetoed by governor)	Mandates coverage; limitations	All health plan enrollees
	2021	HB 1276 (enacted, effective Jan 1, 2023)	Limitations	
Kentucky	2022	HB 58 (not enacted)	Mandates coverage; limitations	All health plan enrollees; includes Medicaid
Massachusetts	2019	S.604 (not enacted)	Limitations	All health plan enrollees
	2021	S.687 (not enacted)		
	2021	H.1060 (not enacted)		
	2023	H.990 (in progress)		
	2023	S.659 (in progress)		
New Hampshire	2021	HB 247 (not enacted)	Mandates coverage; limitations	All health plan enrollees
	2023	HB 554 (in progress)		
Ohio	2019	SB 51 (not enacted)	Mandates coverage	All health plan enrollees; public employees
Pennsylvania	2021	HB 916 (not enacted)	Mandates coverage; limitations	All health plan enrollees; excludes Medicare

Abbreviations: AB, Assembly bill; H, House; HB, House bill; S, Senate.

^a^ Describes content related to coverage of or allowable limitations to coverage for nonpharmacological pain treatments.

^b^ Source: California Health and Safety Code Section 124962.

^c^ Source: Colorado Stat 10-16-104.

**Table 2.** Summary of Dimensions Included in Health Insurance Benefit Mandates for Nonpharmacological Pain Treatments by State

State	California^a^	Colorado^a^	Kentucky	Massachusetts	New Hampshire	Ohio	Pennsylvania
Coverage							
Acupuncture	NS	Required	Required	NA	Required	Required	Required
CBT psychotherapy	NS	NS	Required	NA	Required	NS	NS
Chiropractic	NS	Required	Required	NA	Required	Required	Required
Massage	NS	NS	Required	NA	Required	NS	Required
Occupational therapy	NS	Required	Required	NA	Required	NS	Required
Osteopathic manipulation	NS	NS	Required	NA	Required	Required	Required
Physical therapy	NS	Required	Required	NA	Required	NS	Required
Yoga therapy	NS	NS	NS	NA	Required	NS	NS
Coverage limitations							
Benefit design	NS	At least 6 visits for each covered NPT (enacted)	At least 20 visits; cannot require referral; cannot require use of NPT prior to opioid coverage	Prohibit prior authorization requirements	At least 20 visits; cannot require use of NPT prior to opioid coverage	NPT education required	No annual/ lifetime limits
Cost sharing provisions^b^	NS	Parity with primary care visit (enacted)	Parity with primary care visit	NA	Parity with primary care visit	NS	Parity with primary care visit
Other elements							
Types of pain	NS	Pain diagnosis where opioid might be prescribed (enacted)	Chronic pain	NA	Conditions that cause chronic pain	Acute or chronic pain	Mild to moderate acute/chronic pain
Coverage must be evidence-based	Yes, recommended (enacted)	NS	NS	NA	Yes	Yes	Yes
EHB-related provisions	NS	Requires HHS approval/365 d^c^ (enacted)	NS	NA	NPT defined as rehabilitation/habilitation service^d^	NS	NPT defined as rehabilitation/habilitation service^d^

Abbreviations: CBT, cognitive behavioral therapy; EHB, essential health benefits; NA, not applicable because the legislation is not a mandate; NPT, nonpharmacological pain treatment; NS, not specified.

^a^ Items specified as enacted are related to legislation that is enacted in California (AB 2585) and Colorado (HB 1276). Colorado also introduced and did not enact HB 20-1085.

^b^ Cost sharing for NPTs is not allowed to exceed cost sharing for primary care visits.

^c^ Colorado requires the US Department of Health and Human Services to determine within 365 days if a state benefit mandate exceeds EHBs as defined in the Affordable Care Act, requiring the state to defray the cost.

^d^ New Hampshire and Pennsylvania define nonpharmacological pain treatments as a rehabilitative or habilitative service, one of the categories of benefits required to be covered under the Affordable Care Act.

## Discussion

Recent and ongoing efforts to increase access to, and utilization of, nonopioid alternatives for the treatment of pain have resulted in the introduction of legislation regarding nonpharmacological pain treatments in 7 states. Five recommendations for future legislation to create meaningful and accessible coverage for nonpharmacological pain treatments emerged: (1) include coverage for Medicaid enrollees to advance health equity in access to nonpharmacological pain treatments, (2) require cost-sharing at parity with other primary care visits, (3) cover nonpharmacologic treatments for acute pain in accordance with clinical practice guidelines,^4^ (4) include reference to clinical practice guidelines to ensure consistent and up-to-date coverage, and (5) include language protecting the state from cost liability should the mandate be deemed by the Centers for Medicare and Medicaid Services to exceed EHBs.^6^ Limitations to this study include the potential that relevant legislation was missed due to search term specification error or introduction after August 2023. These findings suggest that states considering enactment of nonpharmacological pain treatment benefit mandates should carefully consider the specificity of the policy language and indicated covered populations to maximize policy effectiveness.

## Notes

Supplement 1. **eAppendix.** Search Terms and Strategies

Supplement 2.
Data Sharing Statement
