# Supplementary material for: Health Insurance Mandates for Nonpharmacological Pain Treatments in 7 US States
Source: JAMA Netw Open. 2024 Apr 10;7(4):e245737. doi: 10.1001/jamanetworkopen.2024.5737 (PMC11007571; doi:10.1001/jamanetworkopen.2024.5737)
Supplement: Supplement 1. — eAppendix. Search Terms and Strategies [file jamanetwopen-e245737-s001.pdf]

Supplemental Online Content

Onstott TN, Hurst S, Kronick R, Tsou A, Groessl E, McMenamin SB. Health insurance mandates for nonpharmacological pain treatments in 7 US states. *JAMA Netw Open.* 2024;7(4):e245737. doi:10.1001/jamanetworkopen.2024.5737

eAppendix. Search Terms and Strategies

This supplemental material has been provided by the authors to give readers additional information about their work.

© 2024 [First author last name and initials or group name] et al. *[Journal name].*

eAppendix. Search Terms and Strategies

Before beginning our analysis, we set parameters for the documents we were interested in. Specific inclusion criteria were that the legislation had to either 1) address health insurer coverage for non-pharmacological pain treatment benefits, 2) specify the terms of health insurance coverage (i.e. cost sharing, prior authorization), or 3) specify the types of providers that insurance must reimburse for providing these services. The initial list was developed by two researchers (TO and AT) searching PoliticoPro and LegiScan, two online legislative tracking websites in November, 2022. A subsequent search was conducted by a third researcher (SM) in August 2023 to identify any additional legislation introduced or enacted in the interim. Terms used in this search included *non-opioid alternative, nonpharmacological pain management treatment, non-opioid pain treatment,* and *opioid addiction AND pain.* We retrieved a total of 18 documents from 11 states. Exclusion criteria were 1) legislation addressing mandated coverage for a single non-pharmacological pain management therapy (e.g. benefit mandate for acupuncture or benefit mandate for chiropractic services) and 2) legislation solely addressing other elements of non-pharmacological pain treatment (i.e. requirements to educate providers or patients on the benefits of non-pharmacological pain treatment). After applying the exclusion criteria we were left with 13 documents from seven states. Final versions of the legislative text were obtained from state legislative websites.

After all documents were collected, each document was reviewed by two researchers (TO, SM). Relevant content was defined *a priori* as being inclusive of content that described the non-pharmacological pain treatment benefit mandate, terms of coverage, or applicable providers. Disagreements in the relevant data selected for analysis were resolved through consensus. The

© 2024 [First author last name and initials or group name] et al. *[Journal name].*

final coded segments from two researchers (TO, SM) were compared in Excel using predetermined thematic categories (“coverage”, “limitations”, and “providers”). Given the amount of variation under the category of “coverage,” this concept was further organized into subcategories, representing the different aspects of coverage policies (“eligible population,” “covered benefits,” and “mandate type”). Similarly, “limitations” was subdivided into 1) limitations related to “cost sharing” and 2) others related to “benefit design.” Finally, we identified three new inductive codes arising from the data analysis: “definition of pain,” “evidence-based,” and “Essential Health Benefits (EHB)-related provisions.” All coding was reviewed by two researchers (TO, SM) and discrepancies in the coding of excerpts were discussed and revised as necessary. Results related to “providers” were not included in the main manuscript.

© 2024 [First author last name and initials or group name] et al. *[Journal name].*
