# Supplementary material for: Health Insurance Mandates for Nonpharmacological Pain Treatments in 7 US States
Source: JAMA Netw Open. 2024 Apr 10;7(4):e245737. doi: 10.1001/jamanetworkopen.2024.5737 (PMC11007571; doi:10.1001/jamanetworkopen.2024.5737)
Supplement: Supplement 2. — Data Sharing Statement [file jamanetwopen-e245737-s002.pdf]

Data Sharing Statement

Onstott. Health Insurance Mandates for Nonpharmacological Pain Treatments in 7 US States. *JAMA Netw Open*. Published April 10, 2024. doi:10.1001/jamanetworkopen.2024.5737

Data
Data available: No

Additional Information
Explanation for why data not available: All data is already publicly available
